# Chronic gamma radiation resistance in fungi correlates with resistance to chromium and elevated temperatures, but not with resistance to acute irradiation

**DOI:** 10.1038/s41598-019-47007-9

**Published:** 2019-08-06

**Authors:** Igor Shuryak, Rok Tkavc, Vera Y. Matrosova, Robert P. Volpe, Olga Grichenko, Polina Klimenkova, Isabel H. Conze, Irina A. Balygina, Elena K. Gaidamakova, Michael J. Daly

**Affiliations:** 1 Center for Radiological Research, Columbia University Irving Medical Center, New York, NY USA; 2 Department of Pathology, Uniformed Services University of the Health Sciences, School of Medicine, Bethesda, MD USA; 3 Henry M. Jackson Foundation for the Advancement of Military Medicine, Bethesda, MD USA; 4 Department of Biology, University of Bielefeld, Bielefeld, Germany; 5 Institute of Medicine and Psychology, Novosibirsk State University, Novosibirsk, Russia; 6 Department of Microbiology and Immunology, Uniformed Services University of the Health Sciences, School of Medicine, Bethesda, MD USA

**Keywords:** Fungi, Systems biology

## Abstract

Exposure to chronic ionizing radiation (CIR) from nuclear power plant accidents, acts of terrorism, and space exploration poses serious threats to humans. Fungi are a group of highly radiation-resistant eukaryotes, and an understanding of fungal CIR resistance mechanisms holds the prospect of protecting humans. We compared the abilities of 95 wild-type yeast and dimorphic fungal isolates, representing diverse *Ascomycota* and *Basidiomycota*, to resist exposure to five environmentally-relevant stressors: CIR (long-duration growth under 36 Gy/h) and acute (10 kGy/h) ionizing radiation (IR), heavy metals (chromium, mercury), elevated temperature (up to 50 °C), and low pH (2.3). To quantify associations between resistances to CIR and these other stressors, we used correlation analysis, logistic regression with multi-model inference, and customized machine learning. The results suggest that resistance to acute IR in fungi is not strongly correlated with the ability of a given fungal isolate to grow under CIR. Instead, the strongest predictors of CIR resistance in fungi were resistance to chromium (III) and to elevated temperature. These results suggest fundamental differences between the mechanisms of resistance to chronic and acute radiation. Convergent evolution towards radioresistance among genetically distinct groups of organisms is considered here.

## Introduction

Across the tree of life, there are dramatic differences in ionizing radiation (IR) resistance. Among organisms from the same order and even between species which share a large core of genes and which evolved from a proximal common ancestor, radiation resistance is not predictable using genomics-based bioinformatic approaches^1–12^. Mounting evidence supports that radioresistance is a polyphyletic metabolic trait that evolved as a byproduct of resistance to other more commonly encountered environmental stressors such as desiccation^11,13–15^. Indeed, the etiologic reactive oxygen species (ROS) responsible for metabolism-induced oxidative stress are the same as those generated from water by IR^10^. It is the relative abundance, distribution and fate of different ROS within and between irradiated species that vary greatly^10,11^. It follows that evolution in an environment that is prone to either biotic or abiotic ROS production^16,17^, could (as a byproduct) drive the evolution of IR-resistant organisms.

As a group, fungi are very IR-resistant and can colonize diverse habitats exposed to harsh conditions, including chronic ionizing radiation (CIR): radioactive waste sites, nuclear disaster zones, and space stations^18–23^. However, not enough is known about fungal stress responses and IR resistance mechanisms. It was recently reported that polyextremotolerant fungi accumulate high concentrations of Mn^2+^ metabolite antioxidant complexes (Mn antioxidants), which very efficiently scavenge IR-induced ROS^11,24,25^. This is analogous to extremely IR-resistant *Deinococcus* bacteria^26,27^. Rationally-designed *Deinococcus* Mn antioxidants are now used in the development of irradiated vaccines and as *in vivo* radioprotectors^28–30^. However, very little is known about the nature of fungal Mn antioxidants^24^. Thus, a study of stress resistance mechanisms in fungi may offer fresh insight into the design of radioprotectors and radiomitigators for humans, and for combatting fungal diseases in humans and in agricultural plants^31,32^. Historically, stress response mechanisms in fungi to chemicals and elevated temperatures have been actively studied in just a few model species (mainly in *Saccharomyces cerevisiae*, *Schizosaccharomyces pombe* and *Candida albicans*)^33–36^. The investigation of radiation responses among fungi is a comparatively neglected topic.

We previously showed that many fungi can grow under intense CIR dose rates of 13–67 Gy/h, with basidiomycete and ascomycete yeasts identified as particularly CIR-resistant groups. Among 145 phylogenetically diverse fungi that we tested, 78 grew under 36 Gy/h^18,37^. Consequently, this dose rate represents a convenient “dividing line” for separating CIR-sensitive from CIR-resistant strains. We also tested the same strains for resistance to acute IR. Unexpectedly, there was only a weak correlation between resistance to CIR and resistance to acute IR^37^. We suggested that the weakness of the association is caused by qualitative differences between chronic and acute radiation stresses: whereas the *rates* of DNA damage production and repair are critical to CIR resistance in replicating cells, the *amounts* of DNA damage (double strand breaks in particular) limit survival in acutely irradiated, non-replicating cells^37,38^.

Here we compared CIR exposure with four *environmentally*-*relevant* stressors by comparing the abilities of 95 yeast or dimorphic fungal strains from two major phyla (*Ascomycota* and *Basidiomycota*) to resist CIR and acute IR; heavy metals; low pH; and elevated growth temperatures. CIR resistance in this study represents the ability to grow (*i*.*e*. to remain metabolically active and proliferate) under continuous irradiation, rather than the ability to remain temporarily dormant during exposure and recover afterwards. The 95 strains (Table 1) were chosen to represent a large and diverse sample of wild-type fungi, which was intended to provide detailed information on stress response ranges. All these strains were able to grow on solid rich medium in the absence of CIR, and then were scored for growth on the same medium incubated under CIR.

**Table 1:** Table of fungal strains and their resistance phenotypes.

Strain #	Name	Phylum	D_10_	CIR growth	lowpH growth	T_max_	HgCl_2_	MER	CrCl_3_	K_2_Cr_2_O_7_
EXF-7729	*Cryptococcus laurentii*	*B*	6.5	1	1	30	10	750	500	25
EXF-3792	*Cryptococcus fonsecae*	*B*	4.2	1	1	39	25	1000	500	50
EXF-6424	*Trichosporon moniliiforme*	*B*	4.1	1	1	39	25	500	750	1000
EXF-6430	*Ustilago davisii*	*B*	4.0	1	1	37	25	2000	3500	100
EXF-5822	*Saccharomyces cerevisiae*	*A*	3.6	1	1	40	25	500	500	100
EXF-6246	*Saccharomyces cerevisiae*	*A*	3.5	0	1	40	25	100	500	100
EXF-5294	*Saccharomyces cerevisiae*	*A*	3.2	0	1	40	25	250	500	10
EXF-5576	*Exophiala dermatitidis A*	*A*	3.0	1	1	45	25	100	1000	500
EXF-5586	*Exophiala dermatitidis B*	*A*	3.0	1	1	45	50	100	1000	250
EXF-5585	*Exophiala dermatitidis C*	*A*	3.0	1	1	50	50	100	750	750
EXF-6408	*Metschnikowia fructicola*	*A*	3.0	1	1	39	10	1000	500	100
EXF-4909	*Saccharomyces bayanus x cerivisiae*	*A*	3.0	1	1	40	25	750	500	500
EXF-5282	*Saccharomyces cerevisiae*	*A*	3.0	1	1	40	10	50	100	10
EXF-5284	*Saccharomyces cerevisiae*	*A*	3.0	1	1	40	25	2500	1000	500
EXF-6248	*Saccharomyces cerevisiae*	*A*	3.0	0	1	37	25	15	500	10
EXF-6761	*Saccharomyces cerevisiae*	*A*	3.0	1	1	40	25	100	500	500
EXF-5295	*Saccharomyces cerevisiae*	*A*	2.8	1	1	40	25	500	500	500
EXF-7173	*Saccharomyces paradoxus*	*A*	2.8	0	1	40	10	50	100	250
EXF-4916	*Saccharomyces cerevisiae*	*A*	2.6	1	1	40	25	1000	500	1000
EXF-5281	*Saccharomyces cerevisiae*	*A*	2.6	1	1	40	10	50	100	250
EXF-5046	*Saccharomyces cerevisiae*	*A*	2.5	1	1	40	25	3000	500	1000
EXF-7200	*Saccharomyces cerevisiae*	*A*	2.5	1	1	40	25	500	500	100
EXF-7135	*Saccharomyces paradoxus*	*A*	2.5	1	1	40	25	50	100	250
EXF-8528	*Rhodotorula nothofagi*	*B*	2.5	0	0	25	25	100	500	10
MD-1149	*Rhodotorula taiwanensis*	*B*	2.5	1	1	32	50	500	500	100
EXF-6676	*Saccharomyces paradoxus*	*A*	2.4	0	1	40	25	50	100	100
EXF-8581	*Rhodotorula fujisanensis*	*B*	2.1	0	0	25	10	100	100	25
EXF-5283	*Metschnikowia pulcherrima*	*A*	2.0	1	1	39	10	1000	750	1000
EXF-6398	*Pichia kudravzevii*	*A*	2.0	1	1	45	25	100	500	750
EXF-5293	*Saccharomyces bayanus*	*A*	2.0	1	1	40	25	3000	500	100
EXF-3422	*Saccharomyces cerevisiae*	*A*	2.0	1	1	40	25	2500	500	500
EXF-4911	*Saccharomyces cerevisiae*	*A*	2.0	1	1	40	25	1000	500	1000
EXF-5042	*Saccharomyces cerevisiae*	*A*	2.0	1	1	40	25	3000	500	500
EXF-5248	*Saccharomyces cerevisiae*	*A*	2.0	1	1	40	25	3000	500	500
EXF-5735	*Saccharomyces cerevisiae*	*A*	2.0	1	1	40	25	1000	500	500
EXF-5872	*Saccharomyces cerevisiae*	*A*	2.0	0	1	40	25	750	500	100
EXF-6247	*Saccharomyces cerevisiae*	*A*	2.0	0	1	40	25	500	100	500
EXF-6684	*Saccharomyces cerevisiae*	*A*	2.0	1	1	40	25	100	500	500
EXF-7284	*Saccharomyces kudriavzevii*	*A*	2.0	0	1	39	10	50	100	250
EXF-308	*Rhodotorula rubra*	*B*	2.0	1	1	37	25	1000	750	100
EXF-6464	*Debaryomyces hansenii*	*A*	1.8	1	1	39	10	100	500	250
EXF-5297	*Saccharomyces cerevisiae*	*A*	1.8	1	1	40	25	1000	100	500
EXF-5875	*Saccharomyces cerevisiae*	*A*	1.8	0	1	40	25	1000	100	500
EXF-7197	*Saccharomyces cerevisiae*	*A*	1.8	1	1	40	25	500	500	100
EXF-7137	*Saccharomyces paradoxus*	*A*	1.8	0	1	40	10	50	100	250
EXF-5043	*Saccharomyces cerevisiae*	*A*	1.6	1	1	40	25	300	100	250
EXF-6789	*Saccharomyces cerevisiae*	*A*	1.6	1	1	40	25	500	500	100
EXF-1630	*Rhodotorula mucilaginosa*	*B*	1.6	1	1	37	25	1000	750	100
EXF-7207	*Saccharomyces kudriavzevii*	*A*	1.5	0	1	39	10	50	100	100
EXF-7211	*Saccharomyces kudriavzevii*	*A*	1.5	0	1	39	10	50	100	250
EXF-7288	*Saccharomyces kudriavzevii*	*A*	1.5	0	1	39	10	50	100	250
EXF-1612	*Rhodosporidium lusitaniae*	*B*	1.5	0	1	25	10	100	500	100
EXF-6410	*Pichia fermentans*	*A*	1.4	1	1	39	10	1000	500	500
EXF-3501	*Rhodosporidium diobovatum*	*B*	1.4	1	1	37	25	1000	750	100
EXF-6402	*Kazachstania exigua*	*A*	1.2	1	1	39	10	100	750	50
EXF-6835	*Saccharomyces cerevisiae*	*A*	1.2	0	1	40	7.5	50	100	750
EXF-7202	*Saccharomyces cerevisiae*	*A*	1.2	1	1	40	25	100	500	100
EXF-3697	*Rhodosporidium kratochvilovae*	*B*	1.2	1	1	37	50	1000	500	250
EXF-8527	*Rhodotorula colostri*	*B*	1.2	0	0	25	25	250	250	100
EXF-6435	*Rhodotorula glutinis*	*B*	1.2	1	1	30	25	1000	500	100
EXF-5870	*Saccharomyces cerevisiae*	*A*	1.1	0	1	40	25	500	500	100
EXF-512	*Rhodosporidium sphaerocarpum*	*B*	1.1	1	1	30	25	1000	500	500
EXF-5557	*Rhodotorula slooffiae*	*B*	1.1	1	1	37	25	100	500	250
EXF-1534	*Rhodotorula lysinophila*	*B*	1.1	1	1	37	25	1000	500	250
EXF-1529	*Rhodotorula minuta*	*B*	1.1	1	1	37	25	1000	500	250
EXF-7977	*Candida sake*	*A*	1.0	1	1	39	10	1000	750	1000
EXF-6453	*Cyberlindnera saturnus*	*A*	1.0	1	1	39	25	500	2000	3500
EXF-1496	*Pichia guilliermondi*	*A*	1.0	1	1	43	25	250	750	1500
EXF-5733	*Saccharomyces cerevisiae*	*A*	1.0	0	1	40	10	3000	500	100
EXF-6780	*Saccharomyces cerevisiae*	*A*	1.0	1	1	40	25	300	100	250
EXF-7163	*Saccharomyces kudriavzevii*	*A*	1.0	0	1	39	10	50	100	250
EXF-7210	*Saccharomyces kudriavzevii*	*A*	1.0	0	1	39	10	50	100	250
EXF-7282	*Saccharomyces kudriavzevii*	*A*	1.0	0	1	39	10	50	100	250
EXF-7289	*Saccharomyces kudriavzevii*	*A*	1.0	0	1	39	10	50	100	250
EXF-6421	*Schwanniomyces pseudopolymorphus*	*A*	1.0	0	0	39	10	1000	500	100
EXF-3409	*Cryptococcus liquefaciens*	*B*	1.0	1	1	39	25	1000	750	50
EXF-513	*Rhodosporidium babjevae*	*B*	1.0	0	1	30	25	1000	500	50
EXF-3661	*Rhodosporidium lusitaniae*	*B*	1.0	0	1	25	25	1500	250	25
EXF-6094	*Rhodotorula calyptogenae*	*B*	1.0	1	1	40	25	1000	500	100
EXF-9815	*Rhodotorula aurantica*	*B*	1.0	0	0	25	2.5	10	100	10
EXF-6425	*Rhodotorula glutinis*	*B*	1.0	1	1	39	50	1000	500	100
EXF-7964	*Wickerhamomyces anomalus*	*A*	0.9	1	1	40	10	1500	3500	1500
EXF-3800	*Rhodotorula benthica*	*B*	0.9	0	1	25	10	1000	500	75
EXF-3909	*Rhodotorula laryngis*	*B*	0.9	0	1	25	25	1000	750	25
EXF-7107	*Geotrichum sp*.	*A*	0.8	1	1	45	10	100	500	100
EXF-5871	*Saccharomyces cerevisiae*	*A*	0.8	1	1	40	25	1000	500	100
EXF-5288	*Kluyveromyces marxianus*	*A*	0.6	1	1	45	10	1000	500	100
EXF-6218	*Saccharomyces cerevisiae*	*A*	0.6	0	1	40	25	500	500	1000
EXF-6219	*Saccharomyces cerevisiae*	*A*	0.6	0	1	40	5	300	100	250
EXF-6436	*Occultifur externus*	*B*	0.6	0	1	37	25	1000	500	250
EXF-6463	*Candida pseudoloambica*	*A*	0.5	1	1	39	10	1500	3500	75
EXF-7145	*Saccharomyces cerevisiae*	*A*	0.5	0	1	40	25	250	100	500
EXF-7167	*Saccharomyces paradoxus*	*A*	0.5	1	1	40	25	50	100	250
EXF-3801	*Rhodosporidium fluviale*	*B*	0.5	0	1	25	25	1000	500	50
EXF-589	*Debaryomyces hansenii*	*A*	0.3	0	1	39	10	100	250	100

Phylum = *A* for *Ascomycota*, *B* for *Basidiomycota*. D_10_ = D_10_ (kGy) in liquid YPD medium. CIRgrowth = Growth (1) at 36 Gy/h in at least one tested solid medium (AM at pH 2.3 or YPD at pH ~6.8), or no growth (0) under these conditions. lowpHgrowth = Growth at pH 2.3 without irradiation. T_max_ = Maximum temperature (°C) that supported growth on solid optimal medium. HgCl_2_, MER, CrCl_3_, K_2_Cr_2_O_7_ = Highest concentrations of these compounds that supported growth (µM) in AM medium at pH ~6.8. The 95 strains were chosen for their ability to grow on solid YPD medium. They are ranked from high to low acute IR resistance (D_10_).

We used logistic regression to quantify the correlations between the growth abilities of studied fungal strains under CIR and their tolerance to the other stressors. Ensemble machine learning, which can handle complex non-linear relationships between variables, was used for additional confirmation of the results. The correlations found in this large number of diverse fungal strains provide new insight and opportunities for additional research into the mechanisms utilized by a multitude of wild-type fungal taxa to counteract severe stresses. In particular, they offer a way forward for studying how and to what extent the molecular mechanisms for resisting CIR are partnered with those for resisting other adverse conditions, and for promoting development of IR countermeasures.

## Methods

### Strains

The 95 basidiomycetous and ascomycetous yeasts in Table 1 were selected from a collection of 145 phylogenetically diverse fungi assembled and reported previously as part of a Department of Energy (DOE) study dedicated to bioremediation of radioactive waste sites^18,37^. We previously showed that many of these fungi could grow under 36 Gy/h^37^. The 95 strains were chosen for their ability to grow well on *solid* YPD medium at room temperature (RT). CIR resistance can be reliably assessed only on solid media, with constant access to atmospheric oxygen from above and nutrients from below. In liquid media the results are poor because the cells locally deplete oxygen (only ~10 ppm available, *vs*. ~200,000 ppm on the surface of solid media) and nutrients and stop growing, and/or radiation-induced mutants can outgrow the wild-type parental strain^27^.

### Resistance measurements for radiation and other stressors

**Figure 1:**
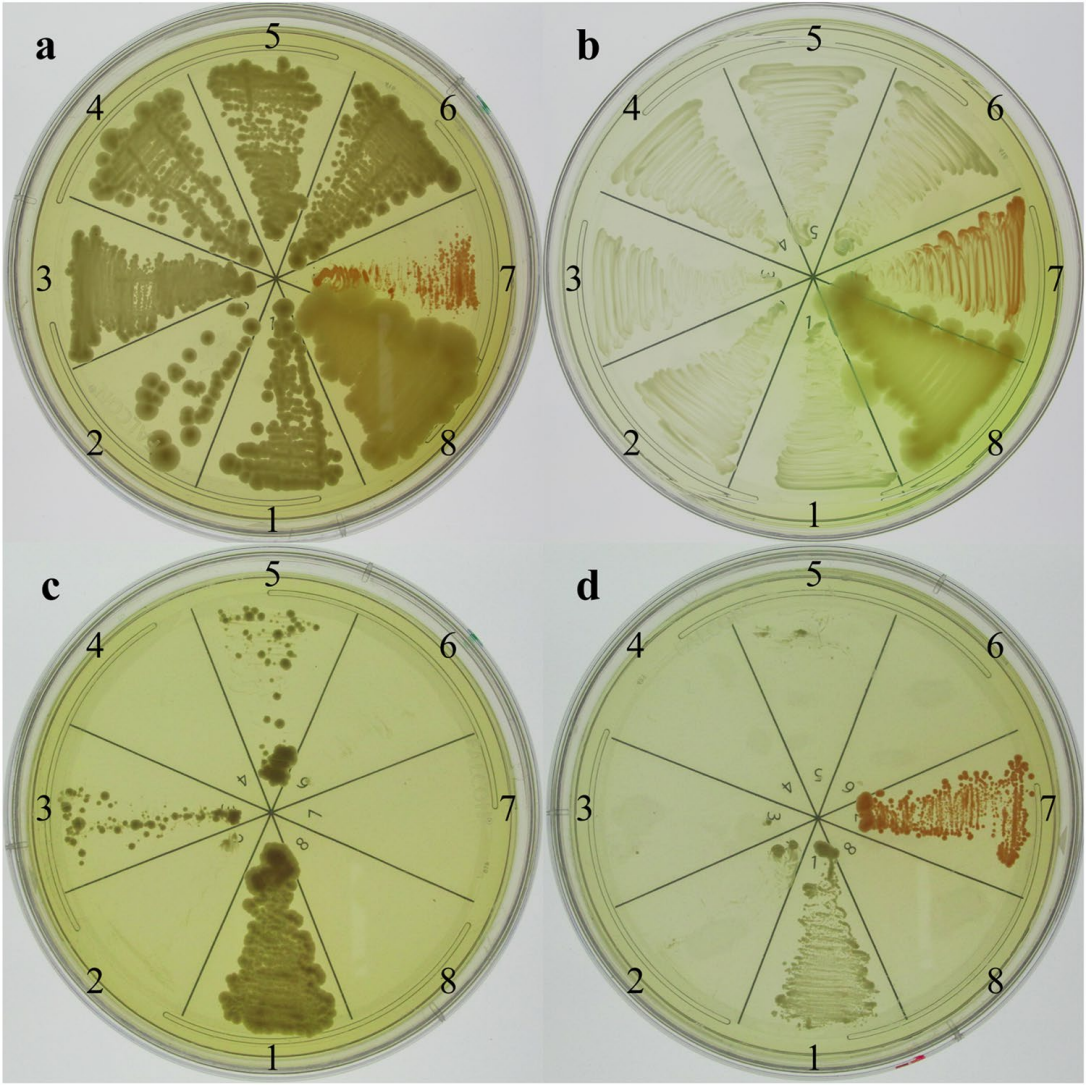
Scoring growth on nutrient agar plates under CIR (36 Gy/h). For a given strain, a sector (1–8) was inoculated on YPD (yeast medium) (**a**,**c**) and TGY (bacterial medium) (**b**,**d**) agar plates. The inoculated plates were incubated in a ^137^Cs gamma irradiator at 22–25 °C. The plates were then photographed and yeast sectors scored as either CIR-resistant (**c**1,3,5) or CIR-sensitive (**c**2,4,6). For each YPD test plate, there was an identically inoculated TGY plate that included two bacteria: *Deinococcus radiodurans* (ATCC BAA-816) (CIR-resistant) and *Pseudomonas putida* (ATCC 47054) (CIR-sensitive), which served as CIR operational controls. Sectors: 1. EXF-6761; 2. EXF-6219; 3. EXF-5735; 4. EXF-6218; 5-6. Standard laboratory *S. cerevisiae* strains FY1679 (diploid) and BY4741 (haploid), respectively; 7. *D*. *radiodurans*; and 8. *P*. *putida*. No irradiation (**a**,**b**); 36 Gy/h (**c**,**d**).

Resistance levels to CIR, acute IR, and heavy metals were measured and compared in 95 different fungal strains listed in Table 1. The ability to grow under CIR (called CIRgrowth, scored as 1 for growth and 0 for no growth) on YPD plates at pH 7.0 (1% yeast extract; 2% peptone; 2% D-glucose; 2% bacteriological agar), was assessed as shown in Fig. 1^18^. Survival following acute forms of gamma radiation was determined on YPD plates by colony forming unit (CFU) assay as described previously^26^, and expressed as the dose killing 90% of the population (D_10_). Acute exposures of liquid fungal cultures were performed in a ^60^Co irradiator (10 kGy/h) at 0 °C.

Throughout this work, chronic exposures (36 Gy/h) were performed in a ^137^Cs irradiator at RT for 3–6 days. This time was sufficient to clearly assess whether or not a given strain is able to grow and proliferate under 36 Gy/h CIR while nutrients in the medium remain abundant. Binary scoring of growth on agar plates was sufficient to perform correlation analysis and created approximately balanced classes containing strains that either did or did not grow.

Maximum growth temperature (T_max_, °C) was determined on solid YPD by inoculating the strain to single colony, incubating the plates at various temperatures (25–50 °C; temperature maxima) for 7 days, and visually inspecting the plates for colony formation. The highest concentrations of HgCl_2_ (Sigma, 215465), merbromin (Sigma, M7011), K_2_Cr_2_O_7_ (Sigma, P2588), and CrCl_3_ (Aldrich, 27096) supporting growth were determined in liquid AM (*Acidiphilium* Medium); YPD is not suitable for measuring metal toxicity because of its metal-chelating properties^18^. The overnight cultures were pre-grown at optimal temperatures in YPD medium, washed twice in sterile MQ and used to inoculate fresh AM media supplemented with different concentrations of heavy metals in 96-well plates to a final OD_600_∼0.1. The strains were incubated at optimal temperatures. After inoculation, the OD_600_ was measured in one week. The lowest pH supporting growth (lowpHgrowth, coded as 0 or 1) was determined as described previously^18^.

The measured variables D_10_, CrCl_3_, HgCl_2_, K_2_Cr_2_O_7_, and MER were log_10_-transformed to bring their distributions closer to normal. This procedure produced the variables called logD_10_, logCrCl_3_, *etc*. Another binary variable called *Ascomycota* was added to test for the potential effect of phylum. It was coded as 1 for strains belonging to *Ascomycota*, and at 0 for strains belonging to *Basidiomycota*.

### Correlation analysis and logistic regression

The associations between the predictor variables (logD_10_, logCrCl_3_, logHgCl_2_, logK_2_Cr_2_O_7_, logMER, T_max_, lowpHgrowth and *Ascomycota*) and the outcome (CIRgrowth) were assessed using *R* 3.5.1 software (https://www.r-project.org/). We calculated Pearson correlation coefficients, and performed logistic regression with information theoretic multi-model inference (MMI) based on the Akaike information criterion with sample size correction (AICc)^39^. The logistic regression with MMI provided a parametric method for assessing the main effects of the predictor variables. As an alternative backup approach that can handle non-linear dependences and is more robust to correlations between predictors^40^, we also analyzed the data using machine learning by customized generalized boosted regression (GB) with synthetic noise variables as benchmarks of predictor performance^41,42^. These machine learning analyses are described in Supplementary Methods.

Pearson correlation matrices of the studied variables were generated using the *cor* and *corrplot* functions in *R* 3.5.1. MMI was implemented using the *glmulti R* package. This approach fitted all possible predictor combinations (model structures) to the data by logistic regression and assessed each combination’s support by AICc. It provided 95% confidence intervals (CIs) for each predictor, taking into account model selection uncertainty (*i*.*e*. the variability in which predictors are present and which are absent from a particular fitted model structure). It also provided a relative importance score for each predictor, which was calculated using the sum of Akaike weights for all fitted model structures that contained the given predictor^39^. The strongest predictors had 95% CIs not overlapping zero and high relative importance scores. They were used to build preferred models, which were then evaluated by constructing receiver operating characteristic (ROC) curves using the *pROC* package.

### Data sets

Logistic regression that included all predictor variables showed that the variable *Ascomycota* produced a high variance inflation factor (VIF) of 4.2, which may indicate multicollinearity in the data set. Consequently, we split the data set into 2 parts by phylum (*Ascomycota*, 67 strains, and *Basidiomycota*, 28 strains) and analyzed these parts separately. Because a substantial number (34) of the studied fungal strains belonged to the species *Saccharomyces cerevisiae*, we tested their influence on the results by performing the analyses after excluding the *S*. *cerevisiae* data, or conversely, by analyzing the *S*. *cerevisiae* data alone.

### Additional analyses

The analysis approaches described above assumed that the data for each fungal strain are statistically independent. To account for the potential effects of correlations between data for different species belonging to the same genus, we also implemented mixed effects logistic regression models with random intercepts and slopes for each genus, using the *lme4 R* package. For the fixed effects, we used each predictor (logD_10_, logCrCl_3_, logHgCl_2_, logK_2_Cr_2_O_7_, logMER, Tmax, lowpHgrowth and *Ascomycota*) one at a time. Several predictors together were not used because such complex mixed effects models did not converge on the data sets used here (yeasts from all phyla combined). Goodness of fit for these mixed effects models was assessed by conditional and marginal R^2^.

## Results

**Figure 2:**
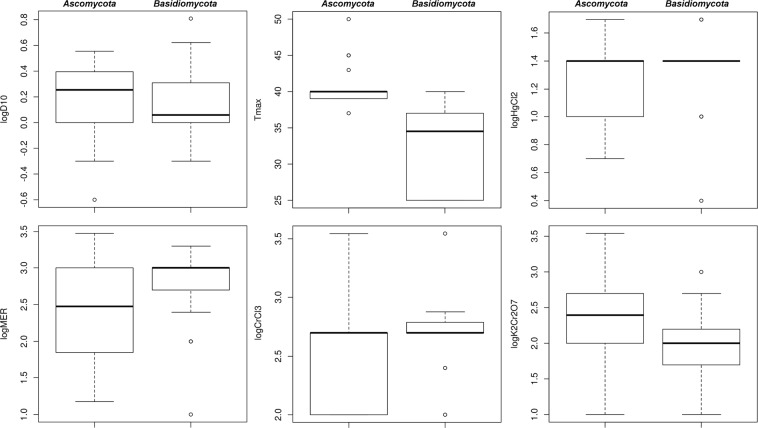
Box plots that summarize and compare continuous predictor variables for yeasts belonging to phylum *Ascomycota* and those belonging to phylum *Basidiomycota*.

**Figure 3:**
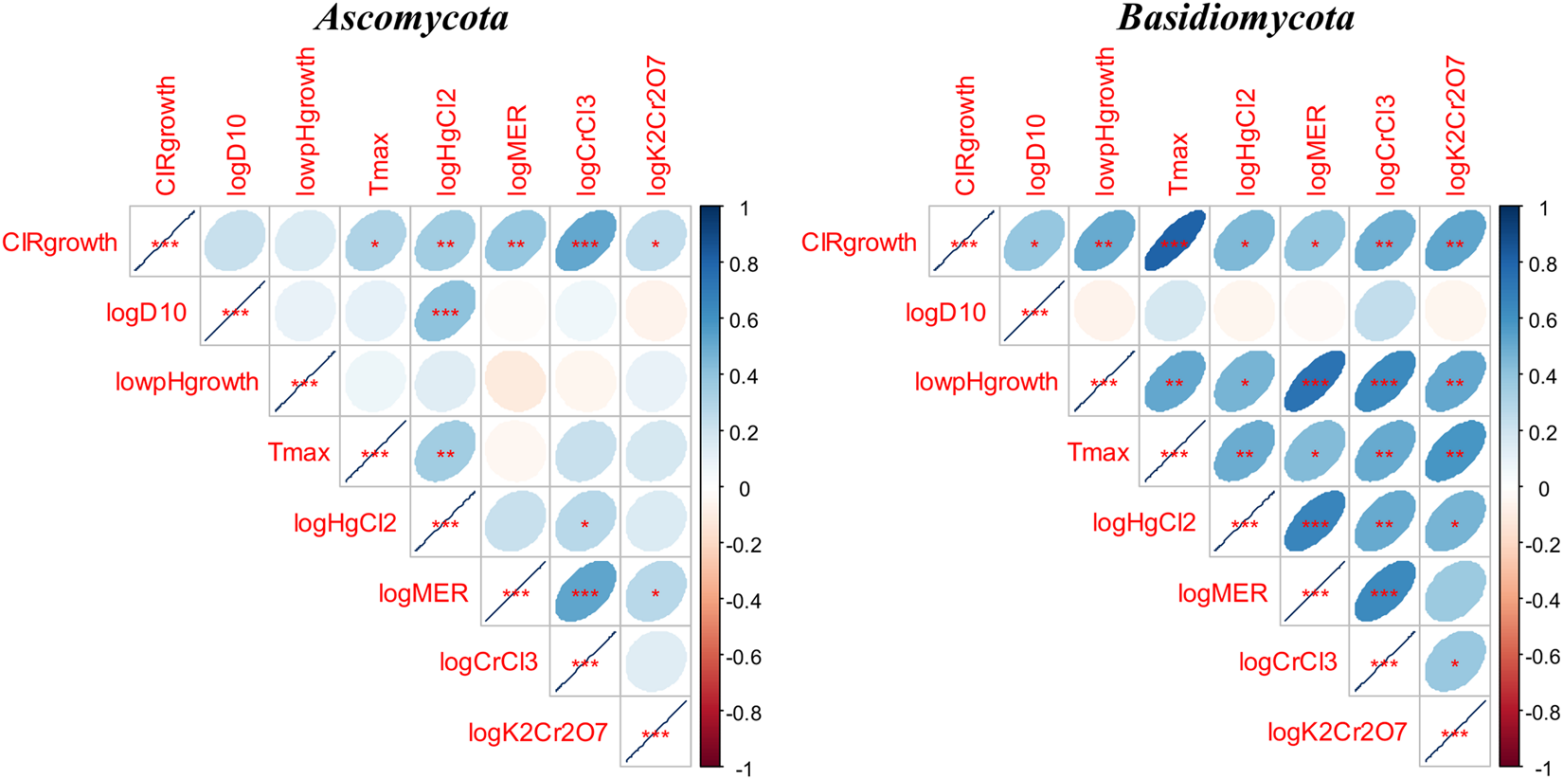
Visualization of pairwise Pearson correlation matrices of all variables for yeasts belonging to phylum *Ascomycota* and those belonging to phylum *Basidiomycota*. Red star symbols indicate statistical significance levels: 3 stars indicate p < 0.001, 2 stars indicate p < 0.01, 1 star indicates p < 0.05, no stars indicates p > 0.05. Due to multiple comparisons, only 3 star significance levels are likely to indicate strong associations. Crossed out boxes represent meaningless correlations of a given variable with itself. Blue ellipses represent positive correlations, and red ones represent negative correlations. Darker color tones and narrower ellipses represent larger correlation coefficient magnitudes.

Visual comparisons of how the continuous predictor variables behaved in each fungal data set are shown in Fig. 2. Correlation matrices of the variables in these data sets are shown in Fig. 3. They suggested that for *Ascomycota* the strongest and most significant correlation of CIRgrowth was with logCrCl_3_, whereas for *Basidiomycota* it was with T_max_. Interestingly, acute IR resistance (logD_10_) did not turn out to be strongly correlated with CIRgrowth (Table 2). For example, several fungi were able to grow under 36 Gy/h CIR despite having logD_10_ below the 25^th^ percentile (EXF-6463 *Candida pseudoloambica*, EXF-7107 *Geotrichum sp*., EXF-5288 *Kluyveromyces marxianus*, EXF-5871 *Saccharomyces cerevisiae*, EXF-7167 *Saccharomyces paradoxus*, EXF-7964 *Wickerhamomyces anomalus*). In contrast, some others were unable to grow under CIR despite having logD_10_ above the 75^th^ percentile (EXF-7173 *Saccharomyces paradoxus* and *S*. *cerevisiae* strains EXF-5294, 6246 and 6248, Supplementary Data File 1). These examples illustrate that discordance between acute IR and CIR resistances can occur even among strains within the same species (*e*.*g*. *S*. *cerevisiae* or *S*. *paradoxus*).

**Table 2:** Associations between various predictor variables and CIR resistance (CIRgrowth, *i*.*e*. ability to grow under 36 Gy/h on YPD).

Predictor	Logistic regression coefficient	95% CI		Relative importance
***Ascomycota***				
logD_10_	0.170	−0.259	0.600	0.521
lowpHgrowth	0.356	−0.517	1.228	0.536
Tmax	0.024	−0.034	0.083	0.544
logHgCl_2_	0.135	−0.289	0.560	0.411
logMER	0.074	−0.122	0.271	0.493
**logCrCl**_**3**_	**0**.**490**	0.181	0.800	0.982
logK_2_Cr_2_O_7_	0.061	−0.121	0.242	0.430
***Basidiomycota***				
logD_10_	0.427	−0.103	0.958	0.831
lowpHgrowth	0.084	−0.201	0.368	0.339
**Tmax**	**0**.**060**	0.035	0.084	1.000
logHgCl_2_	0.029	−0.134	0.192	0.198
logMER	0.010	−0.067	0.087	0.195
logCrCl_3_	0.002	−0.108	0.112	0.183
logK2Cr_2_O_7_	0.035	−0.109	0.178	0.253

These associations were estimated by logistic regression with multi-model inference (MMI) based on the Akaike information criterion with sample size correction (AICc). Those predictor variables that had 95% CIs that did not overlap zero are shown in bold font.

While the correlation matrices show only pairwise correlations, the logistic regression and machine learning approaches looked for associations between CIRgrowth and all predictors analyzed together. These methods agreed with each other, and with the correlation matrices, in identifying logCrCl_3_ as the strongest predictor of CIR growth in *Ascomycota* and T_max_ in *Basidiomycota* (Table 2). Exclusion of *S*. *cerevisiae* did not qualitatively change the results: logCrCl_3_ and T_max_ were still the only predictors that had 95% CIs not overlapping zero in logistic regression MMI analysis and achieved high scores in machine learning analyses (see Supplementary Table 1). Using *S*. *cerevisiae* data alone did not produce strong correlations between any of the variables, perhaps due to a roughly 3-fold reduction in data set size (34 *S*. *cerevisiae* strains vs 95 in the full data set).

**Figure 4:**
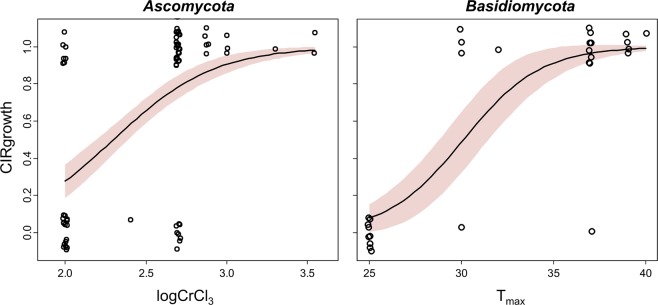
Visualizations of preferred logistic regression models (curves) and data points (open circles) for *Ascomycota* and *Basidiomycota* yeast data sets. Shaded regions around the best-fit model curves represent 95% prediction intervals. The data points had binary values of 0 or 1 on the y-axis (corresponding to no growth or growth under 36 Gy/h, respectively), so to prevent overlap of the data points and improve their visualization we moved them along both the y-axis and the x-axis in these plots by small random increments.

**Figure 5:**
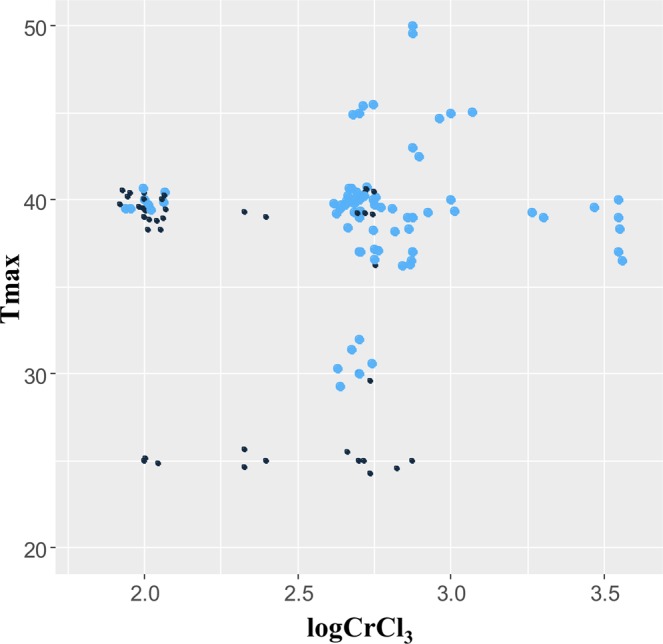
Associations between CIRgrowth with logCrCl_3_ or T_max_ for yeast from both phyla combined. Small black circles represent strains that could not grow under 36 Gy/h (CIRgrowth = 0), and large blue circles represent strains that could grow under this dose rate (CIRgrowth = 1). To prevent overlap of the data points and improve their visualization we moved them along both the y-axis and the x-axis by small random increments.

Based on these results, the preferred logistic regression model for *Ascomycota* contained logCrCl_3_ as the only predictor with a best-fit coefficient of 3.21 (standard error, SE 0.81, p-value 7.66 × 10^−5^) and an intercept value of −7.38 (SE 1.99, p-value 2.12 × 10^−4^). This model achieved good performance in discriminating between strains that were able to tolerate 36 Gy/h from those that were unable to do so: its ROC curve area was 0.795 (95% CI: 0.699, 0.892). The preferred logistic regression model for *Basidiomycota* contained T_max_ as the only predictor with a best-fit coefficient of 0.479 (SE 0.163, p-value 3.26 × 10^−3^) and an intercept value of −14.41 (SE 4.91, p-value 3.37 × 10^−3^). This model achieved very good performance: its ROC curve area was 0.949 (95% CI: 0.862, 1.000). These logistic regression model fits for *Ascomycota* and *Basidiomycota* are shown in Fig. 4. To illustrate the same patterns in a non-parametric way, we also plotted the relationships between CIRgrowth, logCrCl_3_ and T_max_ in Fig. 5.

Combined analyses of yeast data from both phyla with the *Ascomycota* variable removed again pointed to logCrCl_3_ and T_max_ as the best predictors. There was no strong evidence for interactions between logCrCl_3_ and T_max_: when an interaction term between these variables was introduced into logistic regression models, its MMI-corrected 95% CIs overlapped zero.

To account for the potential effects of correlations between data for different species belonging to the same genus, we also implemented mixed effects logistic regression models with random intercepts and slopes for each genus, using combined data for both *Ascomycota* and *Basidiomycota*. For the fixed effects, we used each predictor (logD_10_, logCrCl_3_, logHgCl_2_, logK_2_Cr_2_O_7_, logMER, T_max_, lowpHgrowth and *Ascomycota*) one at a time. The model with T_max_ as the fixed effect predictor outperformed all other tested models and achieved conditional R^2^ (which represents the variance explained by the entire model) of 0.989 and marginal R^2^ (which represents the variance explained by the fixed effects only) of 0.416. In this model, the slope coefficients for T_max_ were smallest for the genera *Cryptococcus* and *Rhodotorula* (1.711 and 1.725, respectively) and largest for the genera *Saccharomyces* and *Schwanniomyces* (2.857 and 2.860, respectively). For the model with logCrCl_3_ as the fixed effect predictor, both the conditional R^2^ and marginal R^2^ were 0.312. These results suggest that the correlation between CIRgrowth and T_max_ varies considerably by genus, whereas the correlation between CIRgrowth and logCrCl_3_ does not appear to vary by genus.

## Discussion

We compared CIR exposure with other stressors by comparing the abilities of 95 yeasts from two different phyla (*Ascomycota* and *Basidiomycota*) (Table 1) to resist the following challenges: (a) Chronic and acute gamma radiation, which generate ROS by water radiolysis^10^. (b) Transition metals that generate ROS by redox-cycling^12^. (c) Low pH (2.3), where protons (H^+^) potentiate the production of hydrogen peroxide (H_2_O_2_) from superoxide (O_2_^.−^)^10^. (d) Elevated growth temperatures that increase production of metabolism-induced ROS^36^. We observed a robust correlation between resistance to CIR and Cr^3+^ (logCrCl_3_) in *Ascomycota* yeasts. The correlation with Cr^6+^ (logK_2_Cr_2_O_7_) was weaker (Fig. 3, Table 2). While Cr^6+^ is a known human carcinogen, its reduction to Cr^3+^ renders the metal less mutagenic and carcinogenic and even essential or beneficial in some situations like diabetes^43,44^. In basidiomycete yeasts, the strongest predictor of CIRgrowth was the maximum temperature that supported growth (T_max_), rather than heavy metal tolerance (Fig. 3, Table 2). All basidiomycete yeast strains with T_max_ below 30 °C were also unable to grow under 36 Gy/h (Fig. 4). Perhaps, this phenomenon is related to antioxidant activity because heat stress is known to enhance ROS production, and counteracting these ROS by antioxidants is involved in yeast thermotolerance^36^.

Significantly, the correlations between all of the tested stressors (CIR, acute IR, low pH, elevated temperature, chromium and mercury) tended to have a positive sign (Fig. 3). This suggests that in yeast some shared mechanisms may contribute to resisting multiple environmental stressors including gamma radiation. For example, two strains of the common laboratory yeast species *S*. *cerevisiae* (EXF-4916 and EXF-5284, Table 2), which is considered an environmentally-robust baker’s yeast^45^, were resistant to all stressors tested (they were at or above the 80^th^ percentile for tolerance to each stressor).

A feature shared by fungi and prokaryotes shown to possess resistance to high radiation doses is their great ROS-scavenging capacity^10,11^. In wild-type yeasts and bacteria, the intracellular content of Mn antioxidants is strongly correlated with IR resistance^11,24^. Indeed, under nutrient-replete conditions, a high intracellular concentration of Mn antioxidants renders antioxidant enzymes such as superoxide dismutase (SOD) dispensable for acute and chronic IR resistance^26,38^. In the case of *Deinococcus spp*., the bacteria are typically capable of surviving 10 kGy of acute radiation under nutrient rich conditions^46^, can grow luxuriantly under CIR at 60 Gy/h, and are resistant to the toxic effects of chromium^19^. Manganese is unique among redox active transition metals found in cells: Mn redox-cycling favors O_2_^.−^ scavenging without the release of extremely reactive hydroxyl (HO•) radicals. In contrast, redox-cycling of other transition metals (*e*.*g*., Fe and Cr) gives rise to HO• radicals (Fenton and Haber-Weiss reactions)^15^. Thus, in cells lacking Mn antioxidants, O_2_^.−^ radicals can become a significant source of HO• radicals, and hence a significant factor in the biochemical mechanism of cellular damage caused by most redox active metals (*e*.*g*. Fe, Cr, U)^10,12,19,46^.

Fungi also accumulate high concentrations of Mn antioxidants and are highly resistant to oxidative stress^11,24^. However, fungal resistance to acute IR (logD_10_) did not turn out to be a good predictor of resistance to CIR (CIRgrowth) in any of the data sets analyzed here (Table 2). Acute IR resistance also appeared to be uncorrelated with the majority of resistances to other tested stressors, except for Hg (logHgCl_2_) in *Ascomycota* (Fig. 3). Lack of correlation between acute IR resistance and resistance to other stressors (including CIR) was also reported in other studies^47,48^, including those on directed evolution of bacteria by selection for acute IR resistance^49^.

Unfortunately, current understanding of chronic radiation resistance and stress responses in general is limited for fungi, outside of a few model species. Our results, based on a large variety of fungi from different phyla, suggest that the mechanisms involved in resistance to acute doses of gamma radiation in fungi may be quite distinct from those involved in resisting the other evaluated stressors, which were chronic by nature. A similar case for bacteria is supported by the finding that the antioxidant enzyme catalase is dispensable for resistance to acute IR, but not CIR^37^; and similarly, *E*. *coli* strains evolved for acute IR resistance by directed evolution can be CIR sensitive^37,49^. A likely explanation is that different responses are needed for cells to cope with large *amounts* of simultaneously produced damage from acute doses of gamma radiation, followed by recovery under non-stressful conditions, compared with those needed to proliferate under a continuously elevated *rate* of damage production (presumably dominated by ROS-related mechanisms) from CIR, heavy metals or elevated temperature^37^.

Our results are consistent with the concept of core stress response, where relatively large numbers of enzymes are expressed in response to different stresses, and exposure to one stress type can cause cross-protection from other stressors^18,33,34^. Core stress responses were investigated and found in several fungal species and are probably widespread, although the specific genes involved can differ widely between species^18,33,34^. Similarly, genetic heterogeneity also is a central characteristic of IR resistance phenotypes in general, which has rendered bioinformatic approaches to gauging ROS stress responses futile^38^. Thus, further physiological studies are warranted to investigate the mechanistic overlap between resistances to CIR, chromium and elevated temperature in certain fungal groups.

## Supplementary information

Supplementary files

Supplementary Dataset 1
