# Supplementary material for: Chronic gamma radiation resistance in fungi correlates with resistance to chromium and elevated temperatures, but not with resistance to acute irradiation
Source: Sci Rep. 2019 Aug 6;9:11361. doi: 10.1038/s41598-019-47007-9 (PMC6684587; doi:10.1038/s41598-019-47007-9)
Supplement: Supplementary file 1 — Supplementary files [file 41598_2019_47007_MOESM1_ESM.pdf]

Chronic gamma radiation resistance in fungi correlates with resistance to chromium and elevated temperatures, but not with resistance to acute irradiation

Igor Shuryak[1*†], Rok Tkavc[2,3*], Vera Y. Matrosova[2,3], Robert P. Volpe[2,3] , Olga Grichenko[2,3], Polina Klimenkova[2,3], Isabel H. Conze[2,4], Irina A. Balygina[2,5], Elena K. Gaidamakova[2,3], Michael J. Daly[2]

*These authors contributed equally to the work

[1]Center for Radiological Research, Columbia University, New York, NY, USA

[2]Department of Pathology, Uniformed Services University of the Health Sciences, School of Medicine, Bethesda, MD, USA

[3]Henry M. Jackson Foundation for the Advancement of Military Medicine, Bethesda, MD, USA

[4]Department of Biology, University of Bielefeld, Bielefeld, Germany

[5] Institute of Medicine and Psychology, Novosibirsk State University, Novosibirsk, Russia

[†] Corresponding author: Igor Shuryak, M.D., Ph.D.

Center for Radiological Research, Columbia University,

630 West 168th street, VC-11-234/5, New York, NY, 10032

Phone: 212-305-2405; Fax: 212-305-3229

E-mail: is144@cumc.columbia.edu

Supplementary Table 1. Associations between various predictor variables and CIR resistance (CIRgrowth, i.e. ability to grow under 36 Gy/h on at least 1 medium) assessed by machine learning. The mean importance (mVIMr) of a given predictor variable, relative to synthetic noise variables, was estimated over 300 generalized boosted regressions with different random number seeds. mVIMr values >1 suggest that the given predictor on average performed better than random noise, whereas values <1 suggest that the predictor's performance was in the noise range. Those predictor variables that had mVIMr >1 are shown in bold font.

**Predictor**	**Mean importance relative to synthetic noise (mVIMr)**	**Standard deviation**
	Ascomycota	
$logD_{10}$	0.292	0.362
lowpHgrowth	0.000	0.000
Tmax	0.454	0.572
$logHgCl_2$	0.358	0.439
logMER	0.760	0.744
**$logCrCl_3$**	**1.746**	0.978
$logK_2Cr2O_7$	0.345	0.402
	Basidiomycota	
$logD_{10}$	0.594	0.834
lowpHgrowth	0.009	0.077
**Tmax**	**6.679**	4.244
$logHgCl_2$	0.176	0.551
logMER	0.035	0.212
$logCrCl_3$	0.424	0.903
$logK2Cr_2O_7$	0.334	0.727

Supplementary Methods

The GB machine learning approach [1], implemented here using the *gbm* package in *R* 3.5.1, uses iterative fitting of decision trees where the data are reweighted during each iteration, focusing the algorithm on fitting those data points on which it performed poorly in previous iterations. Because the outcome variable CIRgrowth was binary, we used the Bernoulli error distribution setting in *gbm*. The other *gbm* parameters like shrinkage (learning rate), n.minobsinnode (minimum number of observations per tree node) and interaction.depth (the maximum variable interaction order) were tuned by trying multiple values of each, evaluating accuracy and performing 10-fold cross validation using the *caret* package.

This GB methodology quantifies the importance (relative influence) of each predictor, but does not provide an easily identifiable "threshold" for distinguishing the most valuable predictors from less useful ones because even completely irrelevant variables with no relationship to the outcome can achieve small, but non-zero, importance scores. An intuitive solution for this problem was proposed by introducing into the data set many synthetic noise variables which serve as convenient "benchmarks" for distinguishing strong from weak predictors [2,3].

This customized GB approach with synthetic noise was implemented as follows, where VIM(X) is the variable importance measure for predictor X, VIM_{min} is the minimum VIM value achieved by the lowest-scoring variable in the data set, $VIM_{NoiseMax}$ is the VIM value achieved by the highest-scoring synthetic noise variable, and VIMr(X) is the VIM of predictor X relative to synthetic noise variables:

$$VIMr(X) = (VIM(X) - VIM_{min})/(VIM_{NoiseMax} - VIM_{min}) \quad (1)$$

VIMr(X) represents an easily interpretable ratio for the performance of predictor X relative to synthetic noise. To generate robust results, we repeated the GB analyses 300 times with different initial random number seeds [4,5], generating a distribution of VIMr values across runs. We used the mean of this distribution, abbreviated as mVIMr, as a threshold for separating strong predictors (with $mVIMr > 1$) from weak ones (with $mVIMr \leq 1$).

We generated 5 synthetic noise variables per each continuous predictor ($logD_{10}$, $logCrCl_3$, $logHgCl_2$, $logK_2Cr_2O_7$, logMER, T_{max}), where each of these noise variables was a vector of normally-distributed random numbers with the same mean and standard deviation as the corresponding predictor. We also randomly perturbed these predictors during each repeat of the machine learning analysis by adding a vector of normally distributed random numbers with a mean of 0 and a standard deviation of 0.05. This was intended to represent realistic error distribution and magnitude values. Larger random number standard deviation values (e.g. 0.1, 0.2) produced qualitatively similar results. For each of the two binary predictors (lowpHgrowth and *Ascomycota*) we also generated 5 synthetic noise variables by permuting the predictors, and did not apply any random noise to the predictor values themselves.

References

1. Hastie, T., Tibshirani, R. & Friedman, J. H. *The elements of statistical learning: data mining, inference, and prediction*. (Springer, 2009). doi:10.1007/BF02985802

2. Shuryak, I. Advantages of Synthetic Noise and Machine Learning for Analyzing Radioecological Data Sets. *PLoS One* **12**, e0170007 (2017).

3. Shuryak, I. Modeling species richness and abundance of phytoplankton and zooplankton in radioactively contaminated water bodies. *J. Environ. Radioact.* **192**, 14–25 (2018).

4. Holzinger, E. R. *et al.* Variable selection method for the identification of epistatic models. pmcid: PMC4299919. *Pac. Symp. Biocomput.* 195–206 (2015).

5. Szymczak, S. *et al.* r2VIM: A new variable selection method for random forests in genome-wide association studies. *BioData Min.* **9**, 7 (2016).
